# ERFMTDA: Predicting tsRNA–disease associations using an enhanced rotative factorization machine

**DOI:** 10.1371/journal.pcbi.1014594

**Published:** 2026-08-18

**Authors:** Wei Lan, Dong Wang, Wenyi Chen, Xuhua Yan, Qingfeng Chen, Shirui Pan, Yi Pan

**Affiliations:** 1 Guangxi Key Laboratory of Multimedia Communications and Network Technology, School of Computer, Electronic and Information, Guangxi University, Nanning, Guangxi, China; 2 Guangxi Key Laboratory of Eye Health, Department of Ophthalmology, the People’s Hospital of Guangxi Zhuang Autonomous Region, Naning, Guangxi, China; 3 School of Information and Communication Technology, Griffith University, Brisbane, Queensland, Australia; 4 Faculty of Computer Science and Control Engineering, Shenzhen University of Advanced Technology, Shenzhen, Guangdong, China; East China JiaoTong University, CHINA

## Abstract

tRNA-derived small RNAs (tsRNAs) have emerged as a novel class of regulatory molecules implicated in the pathogenesis of numerous human diseases, positioning them as promising biomarkers and therapeutic targets. Existing computational methods provide a cost-effective alternative to experimental method, but they tend to ignore biological attributes and complex feature interactions. To overcome these limitations, we propose ERFMTDA, an enhanced rotative factorization machine framework for predicting potential tsRNA-disease associations. ERFMTDA explicitly models complex interactions among heterogeneous biological features while integrating latent structural representations derived from the global association matrix. In addition, a biologically informed negative sampling strategy based on motif-level sequence similarity is introduced to improve the reliability of negative samples. Extensive experiments demonstrate that ERFMTDA consistently surpasses the other eleven state-of-the-art methods. Two case studies on diabetic retinopathy and hepatocellular carcinoma further corroborate the model’s ability to prioritize biologically meaningful tsRNA–disease associations.

## Author summary

tRNA-derived small RNAs (tsRNAs) are a class of regulatory molecules that play important roles in diverse biological processes and human diseases. Identifying disease-associated tsRNAs is crucial for understanding disease mechanisms and developing potential diagnostic and therapeutic strategies. As experimental validation of tsRNA–disease associations is costly and time-consuming, computational methods have become essential for tsRNA–disease association prediction. However, most existing methods focus on network topology and fail to incorporate explicit biological features and complex feature interactions. To address these limitations, we propose ERFMTDA, a computational framework that integrates biological features with global structural information. The framework further incorporates a motif-based negative sampling strategy to reduce false negative samples. By capturing high-order and non-linear interactions among heterogeneous features, ERFMTDA effectively models the complex relationships between tsRNAs and diseases. Extensive validation experiments and two case studies on diabetic retinopathy and hepatocellular carcinoma demonstrate that ERFMTDA can reliably prioritize biologically relevant tsRNA–disease associations, providing a practical tool for guiding future experimental studies.

## Introduction

Non-coding RNAs (ncRNAs) constitute a major component of the human transcriptome and play critical roles in diverse regulatory processes underlying cellular homeostasis and development [1–3]. Among them, tRNA-derived small RNAs (tsRNAs) have emerged as a distinct and functionally versatile class of regulatory molecules, generated through precise cleavage of mature or precursor tRNAs under stress or pathological conditions [4]. In addition, a variety of studies have shown that tsRNAs are associated with multiple human diseases [5–7]. For example, tRF-33 has been identified as a tumor suppressor in gastric cancer. Its dysregulation is closely associated with cancer progression, highlighting its potential as a diagnostic and prognostic biomarker [8]. These findings indicate that tsRNAs are emerging not only as novel regulators of disease processes but also as promising non-invasive diagnostic biomarkers and therapeutic targets.

Despite the growing recognition of their clinical significance, the functional characterization of tsRNAs remains in its infancy compared to other small RNAs. Identifying tsRNA-disease associations through biological experiments is inevitably time-consuming and labor-intensive. Therefore, computational method has become an indispensable strategy for identifying potential disease-related tsRNAs [9,10]. Over the past decade, a variety of computational frameworks have been developed for predicting disease related ncRNAs, including microRNAs (miRNAs), long non-coding RNAs (lncRNAs), and circular RNAs (circRNAs) [11–13]. For instance, Yu et al. [14] proposed a meta-path based method to learns node representations by aggregating information from multiple predefined semantic paths in a miRNA-disease-gene heterogeneous network. Tang et al. [15] developed a Multi-view Multichannel Attention Graph Convolutional Network (MMGCN) to predict potential miRNA-disease associations. Ding et al. [16] designed VGAE-MDA for miRNA-disease association prediction by using dual variational graph auto-encoders on miRNA-based and disease-based sub-networks. Li et al. [17] devised NAGTLDA for lncRNA-disease association prediction by using a node-adaptive graph Transformer that incorporates network structural encoding. Lan et al. [18] proposed GANLDA for lncRNA-disease association prediction by using a graph attention network combined with multi-layer perceptron. Zhao et al. [19] developed MCHNLDA, employing a multi-view contrastive learning framework and a heterogeneous graph attention network with LSTM for lncRNA-disease association prediction. Li et al. [20] introduced Bi-SGTAR, which operates on known circRNA-disease pairs and employs a bi-view sparse gating mechanism to assess the reliability and truthfulness of predicted associations. Yuan et al. [21] proposed iCircDA-NEAE, a method that leverages multiple similarity measures and employs accelerated attribute network embedding with a dynamic convolutional autoencoder to predict circRNA-disease associations. Lan et al. [22] developed LGCDA to predict circRNA-disease associations by fusing local structural features and global similarity features. Liu et al. [23] introduced MPCLCDA, a framework that employs automatic meta-path selection and contrastive learning to derive discriminative features for circRNA-disease association prediction. Lan et al. [24] proposed CLTDA, a contrastive learning–based method that integrates adaptive singular value decomposition and graph convolutional networks for tsRNA–disease association prediction.

The above methods have achieved promising performance in predicting ncRNA–disease associations. But most of them rely heavily on graph structures and similarity-based information, while ignoring explicit biological features and complex feature interactions, thereby limiting their generalization ability for sparse known associations. This limitation is particularly pronounced for tsRNAs, whose functional roles are closely shaped by their biogenesis and sequence characteristics [25,26].

In order to address these limitations, we develop a tsRNA–disease association prediction framework (ERFMTDA) based on Rotative Factorization Machines (RFM) [27]. Specifically, ERFMTDA integrates different sources of information by combining intrinsic biological attributes with global structural representations. The biological attributes of tsRNAs and diseases are first encoded and embedded to preserve domain-specific semantic information. Meanwhile, latent global structural features are extracted from the tsRNA–disease association matrix, allowing the model to capture global association patterns. These heterogeneous features are then fed into a feature interaction learning module to capture high-order dependencies among feature fields, and the resulting interaction representations are further enhanced through a modulus amplification mechanism. In addition, we introduce a motif similarity–constrained negative sampling strategy that avoids selecting diseases associated with similar tsRNAs, thereby improving the reliability of negative samples. Finally, the learned tsRNA–disease pair representation is projected into a scalar score to estimate the probability of their association. To evaluate the effectiveness of ERFMTDA, we conducted extensive experiments including 5-fold and 10-fold cross-validation as well as de novo testing. The results consistently demonstrate that ERFMTDA outperforms other existing methods in predicting tsRNA–disease associations. In addition, two case studies on diabetic retinopathy and hepatocellular carcinoma further confirm that ERFMTDA can effectively identify disease-related tsRNAs in real biological scenarios. The main contributions of this work are summarized as follows:

- We propose ERFMTDA, a novel tsRNA–disease association prediction framework based on Rotative Factorization Machines, which effectively models high-order feature interactions.
- We design a heterogeneous feature integration strategy that combines intrinsic biological attributes with global structural representations, enabling comprehensive characterization of tsRNA–disease associations from heterogeneous information sources.
- We introduce a biologically informed motif similarity–constrained negative sampling strategy to reduce potential false negatives and improve the reliability of negative sample construction.
- Extensive experiments, including 5-fold and 10-fold cross-validation as well as de novo testing, demonstrate that ERFMTDA consistently outperforms existing state-of-the-art methods in predicting tsRNA–disease associations.

## Materials and methods

### Overview

**Fig 1:**
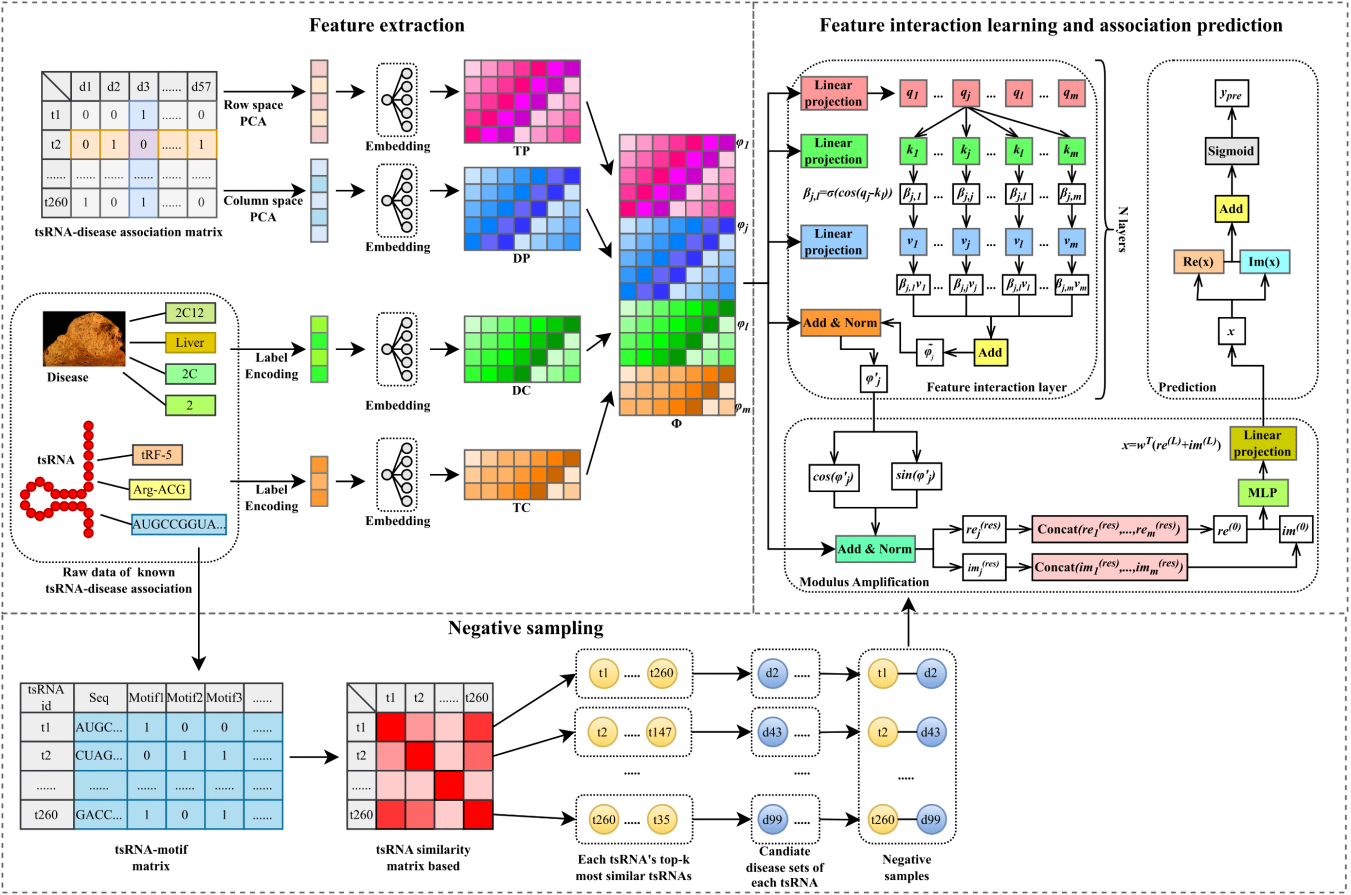
The architecture of ERFMTDA.

The overall workflow of ERFMTDA is summarized in Fig 1, which consists of three stages: (1) feature extraction, (2) feature interaction learning and tsRNA-disease association prediction, (3) negative sampling based on motif similarity.

### Feature extraction

#### Biological feature encoding and embedding.

Each tsRNA contains several intrinsic biological attributes, such as tsRNA type and isotype, etc. To facilitate computational analysis, these attributes are treated as categorical features and encoded using label encoding, where each category is mapped to a unique integer index. As a result, each tsRNA is represented by a discrete feature vector denoted as $\left[{tc}_{1},...,{tc}_{m_{t}}\right]$, where $m_{t}$ represents the number of biological features of each tsRNA and ${tc}_{j}$ is the categorical index corresponding to the *j*-th biological attribute.

Similarly, diseases are characterized by multiple semantic attributes, such as ICD codes and affected organs, etc. These attributes are encoded using the same label encoding strategy to construct a discrete representation for each disease, represented as $\left[{dc}_{1},...,{dc}_{m_{d}}\right]$, where $m_{d}$ denotes the number of semantic features of diseases and ${dc}_{k}$ is the categorical index of the *k*-th disease attribute.

Categorical features are discrete and lack inherent numerical structure, making them difficult to model directly. Therefore, each categorical feature is mapped into a dense continuous space via a learnable embedding lookup operation. Specifically, for the *j*-th biological feature of tsRNAs and the *k*-th semantic feature of diseases, the corresponding embedding vectors are obtained as:


$$\begin{array}{c} {tc}_{j}={𝐄}_{t}^{\left(j\right)}({tc}_{j},:{)}^{⊤}, \end{array}$$ (1)

$$\begin{array}{c} {dc}_{k}={𝐄}_{d}^{\left(k\right)}({dc}_{k},:{)}^{⊤}, \end{array}$$ (2)

where ${𝐄}_{t}^{\left(j\right)}\in {\mathbb{R}}^{N_{j}\times h}$ and ${𝐄}_{d}^{\left(k\right)}\in {\mathbb{R}}^{M_{k}\times h}$ are learnable embedding matrices for the *j*-th biological feature of tsRNAs and the *k*-th semantic feature of diseases, respectively. ${𝐄}_{t}^{\left(j\right)}({tc}_{j},:)$ and ${𝐄}_{d}^{\left(k\right)}({dc}_{k},:)$ denote the rows of the corresponding embedding matrices indexed by ${tc}_{j}$ and ${dc}_{k}$. $N_{j}$ and $M_{k}$ represent the number of categories for each feature, and *h* denotes the dimensionality of the embedding space.

After embedding, each tsRNA and disease are represented as:


$$\begin{array}{c} TC=[{tc}_{1},{tc}_{2},...,{tc}_{m_{t}}]\in {\mathbb{R}}^{h\times m_{t}}, \end{array}$$ (3)

$$\begin{array}{c} DC=[{dc}_{1},{dc}_{2},...,{dc}_{m_{d}}]\in {\mathbb{R}}^{h\times m_{d}}. \end{array}$$ (4)

#### Global structural features extraction.

To capture the global topological structure between tsRNAs and diseases, we define an association matrix $𝐀=(A_{jk})\in {\mathbb{R}}^{n_{t}\times n_{d}}$, where $n_{t}$ and $n_{d}$ denote the number of tsRNAs and diseases, respectively. Each element $A_{jk}$ represents whether the *j*-th tsRNA $t_{j}$ is experimentally verified to be associated with the *k*-th disease $d_{k}$:


$$\begin{array}{c} A_{jk}=\left\{ \begin{array}{cc} 1, & \text{if tsRNA }t_{j}\text{ is associated with disease }d_{k},\\ 0, & \text{otherwise}. \end{array}\right. \end{array}$$ (5)

The *j*-th row of 𝐀 denotes the association profile of tsRNA $t_{j}$ across all diseases, while the *k*-th column represents the association profile of disease $d_{k}$ across all tsRNAs. Since the association matrix is typically high-dimensional and extremely sparse, directly using these profiles may introduce noise and redundancy. Therefore, we apply Principal Component Analysis (PCA) to extract compact global structural representations. PCA is performed on the complete association matrix to preserve the overall topological organization of the tsRNA–disease association space. This design enables the model to capture global structural priors and provides stable unsupervised structural descriptors for all samples.

Specifically, PCA is applied to the row space and column space of the association matrix to capture the principal variation patterns among tsRNAs and diseases, respectively. By projecting the association profiles onto a *u*-dimensional principal subspace, each tsRNA and each disease is represented by a *u*-dimensional global structural feature vector.

For each tsRNA, its global structural features are denoted as $\left[tp_{1},...,tp_{u}\right]$, where $tp_{l}$ represents the *l*-th principal component value. Similar*l*y, for each disease, the global structural features are expressed as $\left[dp_{1},...,dp_{u}\right]$, where $dp_{l}$ denotes the corresponding *l*-th principal component value. Each principal component is treated as an independent feature field that captures a distinct global structural attribute.

To enable effective interaction between global structural features and intrinsic biological attributes, each principal component is further projected into the same *h*-dimensional embedding space. The embedding of the *l*-th structural component for tsRNAs and diseases are defined as:


$$\begin{array}{c} {tp}_{l}={𝐰}_{t}{tp}_{l}+{𝐛}_{t}, \end{array}$$ (6)

$$\begin{array}{c} {dp}_{l}={𝐰}_{d}{dp}_{l}+{𝐛}_{d}, \end{array}$$ (7)

where ${𝐰}_{t}\in {\mathbb{R}}^{h}$ and ${𝐰}_{d}\in {\mathbb{R}}^{h}$ are learnable weight vectors. ${𝐛}_{t}\in {\mathbb{R}}^{h}$ and ${𝐛}_{d}\in {\mathbb{R}}^{h}$ are bias terms. *h* denotes the embedding dimension. The complete global structural embeddings for tsRNAs and diseases are then constructed as:


$$\begin{array}{c} TP=[{tp}_{1},...,{tp}_{u}]\in {\mathbb{R}}^{h\times u}, \end{array}$$ (8)

$$\begin{array}{c} DP=[{dp}_{1},...,{dp}_{u}]\in {\mathbb{R}}^{h\times u}. \end{array}$$ (9)

Finally, the biological, semantic, and global structural feature matrices are assembled into a unified feature representation for each tsRNA–disease pair:


$$\begin{array}{c} \Phi =[TC,DC,TP,DP]=[\phi_{1},...,\phi_{m}]\in {\mathbb{R}}^{h\times m}, \end{array}$$ (10)

where each $\phi_{j}\in {\mathbb{R}}^{h}$ denotes a feature embedding vector, and $m=m_{t}+m_{d}+2u$ is the total number of feature embeddings.

### RFM-based tsRNA-disease association prediction

#### Feature interaction layer.

To capture dependencies among heterogeneous tsRNA and disease features, it is necessary to model interactions among different feature fields. Motivated by a self-attentive rotation mechanism [27], we model dependencies among features based on angular similarity.

For each feature embedding $\phi_{j}$, three linear projections are applied to obtain query, key, and value representations:


$$\begin{array}{c} {𝐪}_{j}={𝐖}_{j}^{Q}\phi_{j},\;{𝐤}_{j}={𝐖}_{j}^{K}\phi_{j},\;{𝐯}_{j}={𝐖}_{j}^{V}\phi_{j}, \end{array}$$ (11)

where ${𝐖}_{j}^{Q}\in {\mathbb{R}}^{h^{\prime }\times h}$, ${𝐖}_{j}^{K}\in {\mathbb{R}}^{h^{\prime }\times h}$, and ${𝐖}_{j}^{V}\in {\mathbb{R}}^{h^{\prime }\times h}$ are learnable, field-specific projection matrices, and $h^{\prime }$ denotes the dimensionality of the projected query, key, and value representations.

To model diverse interaction patterns, a multi-head strategy is employed to split each projected vector into *D* heads:


$$\begin{array}{c} {𝐪}_{j}=[\begin{array}{c} {𝐪}_{j}^{\left(1\right)}\\ \vdots \\ {𝐪}_{j}^{\left(D\right)} \end{array}],\;{𝐤}_{j}=[\begin{array}{c} {𝐤}_{j}^{\left(1\right)}\\ \vdots \\ {𝐤}_{j}^{\left(D\right)} \end{array}],\;{𝐯}_{j}=[\begin{array}{c} {𝐯}_{j}^{\left(1\right)}\\ \vdots \\ {𝐯}_{j}^{\left(D\right)} \end{array}]. \end{array}$$ (12)

For each attention head, the relevance between feature *j* and feature *l* is computed based on angular similarity:


$$\begin{array}{c} s_{j,l}^{\left(d\right)}=\cos({𝐪}_{j}^{\left(d\right)}{)}^{⊤}\cos({𝐤}_{l}^{\left(d\right)})+\sin({𝐪}_{j}^{\left(d\right)}{)}^{⊤}\sin({𝐤}_{l}^{\left(d\right)}), \end{array}$$ (13)

where cos(·) and sin(·) denote the cosine and sine functions, respectively. It enables the model to capture biologically meaningful dependencies, such as the joint contribution of tsRNA-specific attributes and disease-related semantics. The corresponding attention weight is obtained as:


$$\begin{array}{c} \beta_{j,l}^{\left(d\right)}=\sigma (s_{j,l}^{\left(d\right)}), \end{array}$$ (14)

where σ(·) denotes the sigmoid function that maps similarity scores into the interval (0,1) to produce attention weights.

Using these attention weights, the contextualized representation of feature *j* is computed as a weighted aggregation over all feature values:


$$\begin{array}{c} {\tilde{𝐯}}_{j}^{\left(d\right)}=\sum_{l=1}^{m}\beta_{j,l}^{\left(d\right)}{𝐯}_{l}^{\left(d\right)}. \end{array}$$ (15)

The outputs from all heads are concatenated to form the aggregated representation:


$$\begin{array}{c} {\tilde{\phi }}_{j}=Concat({\tilde{𝐯}}_{j}^{\left(1\right)},...,{\tilde{𝐯}}_{j}^{\left(D\right)}), \end{array}$$ (16)

where Concat(·) denotes vector concatenation.

To preserve the original feature information, a residual connection with linear projection is applied:


$$\begin{array}{c} \phi_{j}^{\prime }=LayerNorm({\tilde{\phi }}_{j}+{𝐖}^{R}\phi_{j}), \end{array}$$ (17)

where ${𝐖}^{R}\in {\mathbb{R}}^{h^{\prime }\times h}$ is a shared projection matrix. By stacking multiple such layers, the model progressively refines feature interactions.

#### Modulus amplification.

After the final interaction layer, each refined feature embedding is mapped into the complex plane as follows:


$$\begin{array}{c} {re}_{j}=\cos(\phi_{j}^{\prime }),\;{im}_{j}=\sin(\phi_{j}^{\prime }), \end{array}$$ (18)

where ${re}_{j}$ and ${im}_{j}$ denote the real and imaginary components of the *j*-th feature, respectively.

To preserve first-order feature information, a residual term derived from the original feature embedding is incorporated via a linear projection:


$$\begin{array}{c} {re}_{j}^{\left(res\right)}=LayerNorm({re}_{j}+\cos({𝐖}^{P}\phi_{j})), \end{array}$$ (19)

$$\begin{array}{c} {im}_{j}^{\left(res\right)}=LayerNorm({im}_{j}+\sin({𝐖}^{P}\phi_{j})), \end{array}$$ (20)

where ${𝐖}^{P}\in {\mathbb{R}}^{h^{\prime }\times h}$ is a learnable projection matrix ensuring dimensional consistency.

The resulting complex representations $\{{re}_{j}^{\left(res\right)}+i{im}_{j}^{\left(res\right)}{\}}_{j=1}^{m}$ encode feature interactions in their angular components, while all features lie on the unit circle with fixed magnitude. However, constraining all features to have identical modulus may limit the expressive power of the model.

To address this limitation, the real and imaginary components of all features are concatenated and flattened to form global representations:


$$\begin{array}{c} {re}^{\left(0\right)}=Concat({re}_{1}^{\left(res\right)},...,{re}_{m}^{\left(res\right)})\in {\mathbb{R}}^{mh^{\prime }}, \end{array}$$ (21)

$$\begin{array}{c} {im}^{\left(0\right)}=Concat({im}_{1}^{\left(res\right)},...,{im}_{m}^{\left(res\right)})\in {\mathbb{R}}^{mh^{\prime }}, \end{array}$$ (22)

where $mh^{\prime }$ is the dimensionality obtained by stacking the real and imaginary components of all *m* features. Then they are processed by a shared multilayer perceptron to learn adaptive feature-wise amplitudes:


$$\begin{array}{c} ({re}^{\left(l+1\right)},{im}^{\left(l+1\right)})=ReLU({𝐖}^{\left(l\right)}{re}^{\left(l\right)},{𝐖}^{\left(l\right)}{im}^{\left(l\right)}), \end{array}$$ (23)

where ${𝐖}^{\left(l\right)}$ denote the weight matrix of the *l*-th amplification *l*ayer. This modulus amplification network adaptively rescales the amplitudes of complex feature representations while preserving their angular interaction semantics. In this way, the model can emphasize more informative interaction patterns and suppress less relevant ones, thereby increasing discriminative capacity.

After the final amplification layer, the real and imaginary components are projected onto a scalar:


$$\begin{array}{c} x={𝐰}^{T}({re}^{\left(L\right)}+i{im}^{\left(L\right)}), \end{array}$$ (24)

where *L* denotes the number of amplification layers, 𝐰 is a learnable transformation vector, and *i* is the imaginary unit.

#### Prediction and model training.

The predicted association score between a tsRNA and a disease is obtained as:


$$\begin{array}{c} \hat{y}=\sigma (Re(x)+Im(x)), \end{array}$$ (25)

where σ(·) is the sigmoid function, Re(x) and Im(x) are the real part and imaginary part of *x*, respectively. The resulting score $\hat{y}\in (0,1)$ represents the predicted probability.

The model is trained using binary cross-entropy loss with *L*2 regularization:


$$\begin{array}{c} ℒ(\Theta )=-\frac{1}{N}\sum_{j=1}^{N}[y_{j}\log({\hat{y}}_{j})+(1-y_{j})\log(1-{\hat{y}}_{j})]+\lambda \|\Theta {\|}_{2}^{2}, \end{array}$$ (26)

where $y_{j}$ is the ground truth label. ${\hat{y}}_{j}$ is the predicted value. Θ denotes all trainable parameters, and λ is the *L*2-norm penalty.

### Negative sampling method

Random negative sampling may introduce noisy instances because some randomly selected tsRNA–disease pairs may correspond to undiscovered true associations. Recent studies have demonstrated that tsRNAs exhibit sequence-dependent regulatory activities, including miRNA-like seed-mediated targeting behaviors and motif-associated functional specificity [28–31]. Therefore, we propose a motif similarity–based negative sampling strategy that avoids treating diseases associated with motif-similar tsRNAs as negative samples, thereby reducing the risk of introducing potential false negatives.

#### Motif extraction and binary representation.

For each tsRNA sequence, all motifs with length *L* are extracted. Motifs appearing at least $k^{\prime }$ times are retained to construct a motif vocabulary $V_{m}$. Each tsRNA $s_{j}$ is represented as a binary motif vector:


$$\begin{array}{c} {𝐦}_{j}=[m_{j,1},m_{j,2},...,m_{j,|V_{m}|}]\in \{0,1{\}}^{\left|V_{m}\right|}, \end{array}$$ (27)

where


$$\begin{array}{c} m_{j,k}=\left\{ \begin{array}{cc} 1, & \text{if motif }v_{k}\in V_{m}\text{ occurs in }s_{j},\\ 0, & \text{otherwise}. \end{array}\right. \end{array}$$ (28)

By stacking all tsRNA motif vectors, we obtain the binary motif matrix:


$$\begin{array}{c} 𝐌=[\begin{array}{c} {𝐦}_{1}\\ {𝐦}_{2}\\ \vdots \\ {𝐦}_{n_{t}} \end{array}]\in \{0,1{\}}^{n_{t}\times |V_{m}|}, \end{array}$$ (29)

which captures the occurrence patterns of all motifs across tsRNAs.

#### Motif-based similarity computation.

Based on 𝐌, motif similarity between tsRNAs is computed using cosine similarity:


$$\begin{array}{c} S_{jk}=\frac{m_{j}\cdot m_{k}}{{\left\|m_{j}\right\|}_{2}{\left\|m_{k}\right\|}_{2}}, \end{array}$$ (30)

where a higher value of $S_{jk}$ indicates greater motif composition similarity between tsRNA $t_{j}$ and $t_{k}$.

#### Similarity-constrained negative sampling.

For each tsRNA $t_{j}$, the top-*k* most similar tsRNAs are collected into a set denoted as ${𝒮}_{j}^{t}$. To avoid selecting diseases that are already associated with any tsRNA in ${𝒮}_{j}^{t}$, we construct a forbidden set of diseases defined as:


$$\begin{array}{c} {𝒮}_{j}^{d}=\underset{t_{r}\in \{t_{j}\}\cup {𝒮}_{j}^{t}}{⋃}\{d_{k}\;|\;A_{rk}=1\}, \end{array}$$ (31)

where $A_{rk}=1$ indicates a known association between tsRNA $t_{r}$ and $d_{k}$.

The candidate disease set for $t_{j}$ is then expressed as:


$$\begin{array}{c} C_{j}=\{d_{k}\;|\;d_{k}\notin {𝒮}_{j}^{d}\}. \end{array}$$ (32)

A disease $d_{k}$ is randomly selected from $C_{j}$ to form a negative pair ($t_{j},d_{k}$). This procedure is repeated until the number of negative pairs equals that of the positive pairs, ensuring that the generated negative samples have a higher probability of representing true negatives rather than unverified positives.

## Results

### Dataset

To ensure high data quality and experimental reliability, we constructed a curated tsRNA–disease association dataset through a systematic literature search in PubMed. Specifically, publications indexed up to December 2025 were retrieved using the keywords “tsRNA”, “tRF”, “tRNA-derived fragments”, and “disease”, followed by manual screening of the retrieved studies.

To guarantee the reliability of the collected associations, we applied strict inclusion criteria and retained only experimentally validated tsRNA–disease pairs supported by reverse transcription quantitative PCR (RT-qPCR) evidence. During preprocessing, tsRNA names were standardized using tDRnamer (version 1.2) [32], a dedicated nomenclature framework for systematic annotation of tRNA-derived small RNAs. Disease names were unified by merging synonymous terms and resolving naming inconsistencies across different studies. Duplicate association records reported in multiple publications were removed to avoid redundancy.

After applying these preprocessing and filtering procedures, the final benchmark dataset contained 260 unique tsRNAs, 57 diseases, and 305 experimentally confirmed associations. The resulting categorical feature space comprised 7 distinct tsRNA types, 44 isotypes, 7 ICD code categories, and 32 affected-organ annotations, which define the input dimensions for the corresponding label-encoding and embedding operations.

### Evaluation metrics

To comprehensively assess the predictive performance of ERFMTDA, we performed 5-fold and 10-fold cross-validation, as well as de novo testing.

To quantitatively evaluate the model, we adopt six widely used metrics: Area Under the Receiver Operating Characteristic Curve (AUC), Area Under the Precision–Recall Curve (AUPR), Accuracy (Acc), Precision (Pre), Recall (Rec), and F1-score. Let TP, TN, FP, and FN denote true positives, true negatives, false positives, and false negatives, respectively. These metrics are defined as follows:


$$\begin{array}{c} Acc=\frac{TP+TN}{TP+TN+FP+FN}, \end{array}$$ (33)

$$\begin{array}{c} Pre=\frac{TP}{TP+FP}, \end{array}$$ (34)

$$\begin{array}{c} Rec=\frac{TP}{TP+FN}, \end{array}$$ (35)

$$\begin{array}{c} F1=\frac{2\times Pre\times Rec}{Pre+Rec}, \end{array}$$ (36)

$$\begin{array}{c} TPR=\frac{TP}{TP+FN}, \end{array}$$ (37)

$$\begin{array}{c} FPR=\frac{FP}{FP+TN}. \end{array}$$ (38)

The ROC curve is constructed by plotting the true positive rate (TPR) against the false positive rate (FPR) under varying classification thresholds, and its area (AUC) reflects the model’s overall discriminative ability. The precision–recall (PR) curve plots Precision versus Recall across thresholds, and its area (AUPR) characterizes the balance between these two quantities. Together, these metrics provide a multidimensional evaluation of ERFMTDA’s predictive performance.

### Parameter settings

The key hyperparameters are set as follows: the dimension of feature embeddings and the hidden units in the attention layers are both set to 32. To mitigate overfitting, a dropout rate of 0.1 is applied to both the attention mechanism and the amplification network. The model is optimized using the Adam [33] optimizer with a learning rate of $1\times 10^{-3}$. The weight decay coefficient of *L*2 regularization is set to $1\times 10^{-5}$. The batch size is set to 32, and the training process is conducted for 200 epochs. For motif extraction, the minimum occurrence threshold $k^{\prime }$ is set to 3.

### Baseline models

To the best of our knowledge, only a limited number of computational models have been specifically developed for tsRNA–disease association prediction. Therefore, we select representative ncRNA–disease association prediction methods as baselines for comparison. Although most of these methods are originally proposed for circRNA–disease association prediction, these approaches are methodologically transferable to the tsRNA–disease prediction task because they are designed to predict latent interactions between non-coding RNAs and diseases. Moreover, the selected baselines provide broad methodological coverage, including random walk–based methods, matrix factorization approaches, graph regularization models, and deep representation learning frameworks. This makes them appropriate and informative benchmarks for evaluating the effectiveness of ERFMTDA. The selected methods are as follows:

- **IBNPKATZ** [34] predicts circRNA–disease associations by combining bipartite network projection and the KATZ measure based on known associations and similarity information.
- **RWR** [35] infers potential circRNA–disease associations via random walk with restart on circRNA and disease similarity networks constructed using Gaussian interaction profiles.
- **iCircDA-MF** [36] integrates circRNA and disease similarities derived from semantic information and known circRNA–gene–disease associations, followed by neighborhood correction and matrix factorization to estimate association scores.
- **LLCDC** [37] reconstructs circRNA and disease similarity networks using locality-constrained linear coding, and subsequently performs label propagation on the integrated networks.
- **DWNN-RLS** [38] employs Kronecker product kernels constructed from circRNA and disease similarities, together with regularized least squares and decreasing-weight k-nearest neighbor initialization, to predict associations.
- **RWRKNN** [39] predicts circRNA–disease associations by combining random walk with restart to capture global network topology and k-nearest neighbors for association classification.
- **GMNN2CD** [40] predicts circRNA–disease associations by learning deep representations from integrated circRNA and disease similarities using a graph Markov neural network with variational and label propagation autoencoders.
- **CD-LNLP** [41] predicts circRNA–disease associations by applying label propagation on circRNA–circRNA and disease–disease similarity graphs derived from known association profiles and integrating the two prediction results.
- **KATZHCDA** [42] predicts circRNA–disease associations by applying KATZ measures on a heterogeneous network constructed from circRNA expression profiles, disease phenotype similarity, and Gaussian interaction profile kernels.
- **RNMFLP** [43] predicts circRNA–disease associations by updating the association matrix using integrated similarity information, followed by robust nonnegative matrix factorization and label propagation.
- **DMFCDA** [44] predicts circRNA–disease associations by learning latent representations of circRNAs and diseases through deep matrix factorization that incorporates both explicit and implicit feedback.

All baseline models were carefully adapted to the tsRNA–disease association prediction task following their original methodological formulations. To ensure a fair comparison, hyperparameters were systematically tuned on the current benchmark dataset according to the settings reported in the corresponding studies whenever applicable.

### Five-fold cross validation

**Fig 2:**
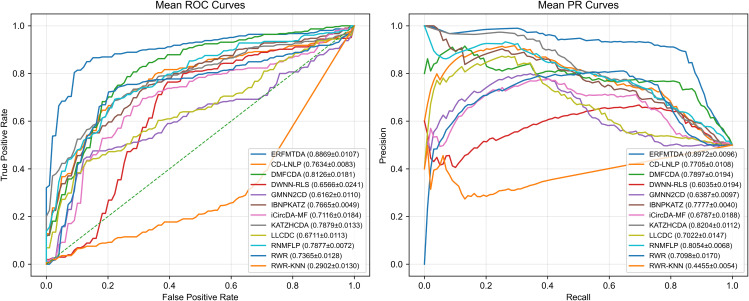
ROC curves and PR curves of ERFMTDA and eleven baseline methods under 5-fold cross-validation.

To assess the robustness of ERFMTDA, we first conduct 10 independent runs with different random seeds under the five-fold cross-validation setting, and the detailed performance statistics are summarized in S1 Table. Then we compare ERFMTDA with all baseline models under the same settings to ensure a fair and consistent evaluation. As illustrated in Fig 2, ERFMTDA achieves the best overall performance among all competing methods, with an average AUC of 0.8869 and an AUPR of 0.8972.

When compared with representative path-based and propagation-based methods, ERFMTDA shows a substantial performance improvement. Specifically, relative to KATZHCDA, which achieves the best performance within this category, ERFMTDA improves AUC and AUPR by approximately 12.6% and 9.4%, respectively. This indicates that modeling heterogeneous feature interactions provides stronger predictive capability than relying solely on network topology and path-based similarity propagation.

Compared with matrix factorization–based approaches, ERFMTDA also demonstrates clear advantages. In comparison with DMFCDA, the strongest matrix factorization baseline, ERFMTDA achieves a 9.1% improvement in AUC and a 13.6% improvement in AUPR. This suggests that ERFMTDA is more effective at capturing complex nonlinear relationships between tsRNAs and diseases, rather than projecting associations into a shared low-dimensional latent space.

In addition, ERFMTDA significantly outperforms kernel-based and deep learning–based methods, such as DWNN-RLS and GMNN2CD. The inferior performance of these methods may be attributed to their heavy dependence on similarity quality and network structure, which can be severely affected by data sparsity in tsRNA–disease association datasets.

Notably, the performance of ERFMTDA are particularly pronounced in terms of AUPR, highlighting its superior ability to identify tsRNA–disease associations under sparsely dataset. Table 1 further summarizes the results of additional evaluation metrics, including Precision, Recall, Accuracy, and F1-score, where ERFMTDA consistently achieves the best performance. These results collectively demonstrate that ERFMTDA provides more accurate and reliable predictions than existing methods for tsRNA–disease association prediction.

**Table 1:** Performance comparison of ERFMTDA with eleven baseline methods under 5-fold cross-validation.

Method	Acc	Pre	Rec	F1
IBNPKATZ	0.7125±0.0102	0.7189±0.0103	0.6977±0.0108	0.7081±0.0105
RWR	0.7461±0.0170	0.7539±0.0174	0.7305±0.0174	0.7420±0.0174
iCircDA-MF	0.7108±0.0154	0.7180±0.0160	0.6944±0.0154	0.7060±0.0157
LLCDC	0.6223±0.0062	0.6264±0.0064	0.6059±0.0062	0.6160±0.0063
DWNN-RLS	0.6656±0.0170	0.6712±0.0175	0.6492±0.0170	0.6600±0.0173
RWR-KNN	0.3805±0.0373	0.3764±0.0386	0.5603±0.1434	0.4367±0.0733
GMNN2CD	0.6540±0.0050	0.7695±0.0133	0.4413±0.0066	0.5590±0.0061
CD-LNLP	0.7177±0.0112	0.6982±0.0102	0.7669±0.0112	0.7309±0.0107
KATZHCDA	0.7440±0.0127	0.7493±0.0123	0.7328±0.0147	0.7409±0.0133
RNMFLP	0.7303±0.0100	0.7298±0.0099	0.7315±0.0103	0.7306±0.0101
DMFCDA	0.7718±0.0193	0.7481±0.0199	0.8226±0.0282	0.7825±0.0186
ERFMTDA	**0.8259±0.0120**	**0.8060±0.0128**	**0.8612±0.0155**	**0.8320±0.0118**

**Note**: Pre, Rec, Acc, and F1 of different methods in the 5-fold cross validation experiment. The best performance is in bold.

### Ten-fold cross validation

**Fig 3:**
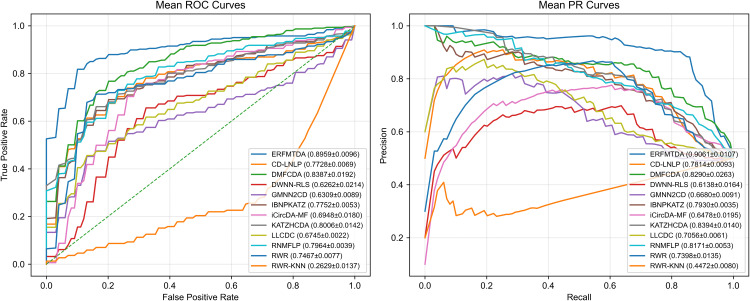
ROC curves and PR curves of ERFMTDA and eleven baseline methods under 10-fold cross-validation.

To further evaluate the robustness of ERFMTDA, we additionally conduct 10 independent runs with different random seeds under the ten-fold cross-validation setting, and the detailed performance statistics are summarized in S2 Table. As shown in Fig 3, ERFMTDA consistently achieves the best overall performance among all competing approaches, with an average AUC of 0.8959 and an AUPR of 0.9061.

Notably, the performance trends observed under the ten-fold cross-validation setting are highly consistent with those obtained from five-fold cross-validation. ERFMTDA maintains clear advantages across all evaluation metrics, including Precision, Recall, Accuracy, and F1-score, as summarized in Table 2. Moreover, the AUC and AUPR values obtained under the two cross-validation strategies remain highly comparable, with only minor variations between the repeated-run averages. This further indicates that the predictive performance of ERFMTDA is stable and not sensitive to the choice of data partitioning.

**Table 2:** Performance comparison of ERFMTDA with eleven baseline methods under 10-fold cross-validation.

Method	Acc	Pre	Rec	F1
IBNPKATZ	0.7261±0.0054	0.7336±0.0055	0.7103±0.0057	0.7218±0.0056
RWR	0.7420±0.0113	0.7496±0.0117	0.7265±0.0113	0.7379±0.0115
iCircDA-MF	0.7003±0.0161	0.7071±0.0167	0.6838±0.0161	0.6953±0.0164
LLCDC	0.6358±0.0056	0.6405±0.0058	0.6194±0.0057	0.6297±0.0057
DWNN-RLS	0.6560±0.0133	0.6613±0.0137	0.6395±0.0133	0.6502±0.0135
RWR-KNN	0.3045±0.0321	0.2979±0.0331	0.3604±0.0840	0.3174±0.0500
GMNN2CD	0.6594±0.0052	0.7809±0.0116	0.4474±0.0057	0.5648±0.0071
CD-LNLP	0.7260±0.0106	0.7057±0.0096	0.7753±0.0106	0.7389±0.0101
KATZHCDA	0.7517±0.0121	0.7591±0.0131	0.7377±0.0114	0.7482±0.0120
RNMFLP	0.7375±0.0077	0.7368±0.0078	0.7390±0.0078	0.7379±0.0077
DMFCDA	0.7840±0.0206	0.7607±0.0271	0.8400±0.0234	0.7953±0.0176
ERFMTDA	**0.8298±0.0141**	**0.8108±0.0179**	**0.8673±0.0159**	**0.8363±0.0140**

**Note**: Pre, Rec, Acc, and F1 of different methods in the 10-fold cross validation experiment. The best performance is in bold.

These consistent results across different cross-validation schemes further demonstrate the robustness and reliability of ERFMTDA, confirming that its superior performance is not attributable to a particular data split but rather to its effective modeling of feature interactions in tsRNA–disease association prediction.

### Ablation experiment

To investigate the individual contributions of the global structural features and the negative sampling strategy based on motif similarity, we construct several ablated variants of ERFMTDA. Each variant removes one or both key components of the proposed model, as described below:

- **ERFMTDA/-pca** removes the global structural features, allowing us to assess the contribution of global structural information.
- **ERFMTDA/-ns** discards the motif-similarity-based negative sampling strategy and adopts random negative sampling, evaluating the effect of negative sample construction.
- **ERFMTDA/-pca,-ns** removes both the global structural features and the motif-similarity-based negative sampling strategy, serving as a simplified baseline model.
- **ERFMTDA** represents the complete model that incorporates all proposed components.

**Fig 4:**
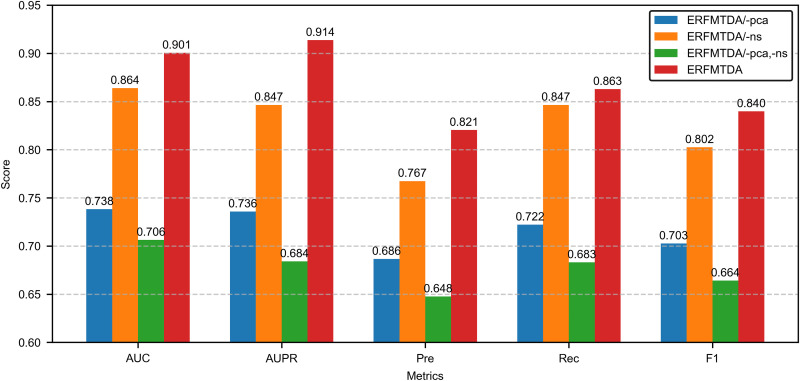
Performance comparison of ERFMTDA and its ablated variants.

The performance of all variants is illustrated in Fig 4, where multiple evaluation metrics, including AUC, AUPR, Precision, Recall, and F1-score, are presented in a bar chart. These results lead to the following observations.

Compared with ERFMTDA/-pca,ns, both ERFMTDA/-pca and ERFMTDA/-ns exhibit substantial performance improvements. In particular, ERFMTDA/-ns achieves an AUC of 0.8637, surpassing ERFMTDA/-pca,ns (AUC = 0.7066) by 22.30%. These results indicate that both the global structural features and the motif-based negative sampling strategy contribute positively to the overall performance of the model. Furthermore, the full model ERFMTDA consistently outperforms its simplified variants. ERFMTDA achieves the highest AUC (0.9004), AUPR (0.9138), Precision (0.8205), Recall (0.8628), and F1-score (0.8398). These results demonstrate that combining global structural features with motif-similarity-based negative sampling yields the most significant performance improvement.

### Effectiveness analysis of the negative sampling strategy

Random negative sampling may introduce instability due to its inherent randomness, potentially leading to performance variations across different runs. To further evaluate the effectiveness and reliability of the proposed motif-similarity-based negative sampling strategy, we conduct six independent experiments for both ERFMTDA and ERFMTDA/-ns, and report the averaged evaluation metrics in Table 3. The full model achieves an average AUC of 0.8858, AUPR of 0.8933, Precision of 0.7986, Recall of 0.8639, and F1-score of 0.8289, all of which are consistently higher than the corresponding averages of ERFMTDA/-ns. These results provide strong evidence that the motif-similarity-based negative sampling strategy effectively enhances model performance.

**Table 3:** Average performance of ERFMTDA and ERFMTDA/-ns over six independent experiments.

Method	Seed	AUC	AUPR	Pre	Rec	F1
ERFMTDA/-ns	7	0.8619	0.8347	0.7872	0.8825	0.8303
ERFMTDA/-ns	42	0.8622	0.8618	0.7735	0.8464	0.8081
ERFMTDA/-ns	512	0.8557	0.8539	0.7820	0.8693	0.8229
ERFMTDA/-ns	1024	0.8678	0.8727	0.7640	0.8336	0.7957
ERFMTDA/-ns	1983	0.8639	0.8465	0.7674	0.8465	0.8025
ERFMTDA/-ns	2025	0.8766	0.8910	0.8048	0.8660	0.8335
ERFMTDA/-ns(mean)	/	0.8647	0.8601	0.7798	0.8574	0.8155
ERFMTDA	7	0.9122	0.9152	0.8258	0.8792	0.8511
ERFMTDA	42	0.8845	0.9023	0.8118	0.8823	0.8453
ERFMTDA	512	0.8731	0.8812	0.7562	0.8594	0.8037
ERFMTDA	1024	0.8670	0.8547	0.7794	0.8631	0.8177
ERFMTDA	1983	0.9006	0.9138	0.8205	0.8628	0.8398
ERFMTDA	2025	0.8776	0.8928	0.7978	0.8366	0.8156
ERFMTDA(mean)	/	**0.8858**	**0.8933**	**0.7986**	**0.8639**	**0.8289**

**Note**: The mean performance of ERFMTDA/-ns is underlined, while the mean performance of ERFMTDA is highlighted in bold.

### De novo experiment

To further evaluate the model’s ability to predict associations involving previously unseen diseases, we conduct a de novo experiment. In this setting, each disease is treated as a “new” disease in turn. Specifically, for a given disease, all known associations between that disease and tsRNAs are removed from the training set. The model is then trained on the remaining data and used to predict the associations between the held-out disease and all tsRNAs. This procedure is repeated for every disease, and the final performance is reported as the average across all runs.

**Fig 5:**
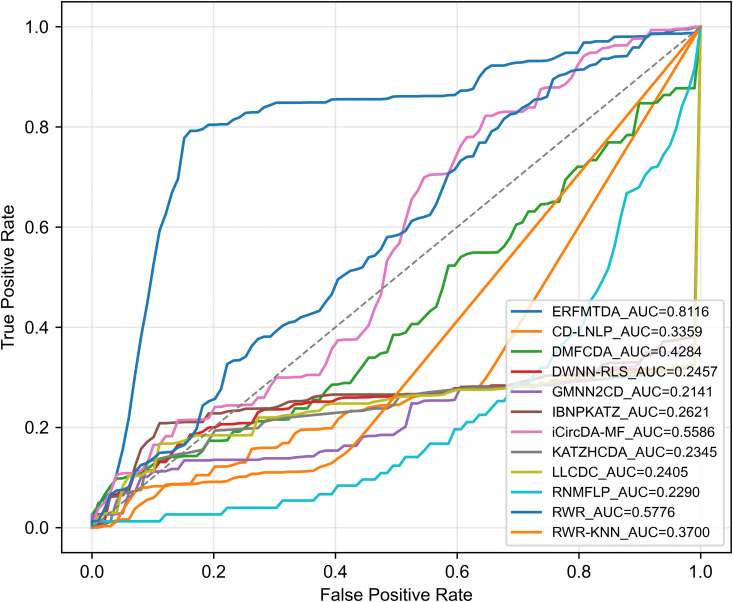
ROC curves of ERFMTDA and other baseline methods in the denovo experiment.

Similar to the five-fold cross-validation experiment, we compare ERFMTDA with the other eleven baseline methods. As illustrated in Fig 5, ERFMTDA achieves an AUC of 0.8116, substantially outperforming all competing approaches. The detailed evaluation metrics are summarized in Table 4. ERFMTDA attains an AUPR of 0.1149, a Precision of 0.0358, a Recall of 0.8596, and an F1-score of 0.0646, all of which are significantly higher than those of the baseline methods.

**Table 4:** Performance comparison of ERFMTDA and other baseline methods in the denovo experiment.

Method	AUPR	Pre	Rec	F1
IBNPKATZ	0.0398	0.0075	0.2656	0.0137
RWR	0.0548	0.0240	0.5810	0.0433
iCircDA-MF	0.0478	0.0139	0.5633	0.0262
LLCDC	0.0388	0.0069	0.2615	0.0127
DWNN-RLS	0.0303	0.0074	0.2622	0.0135
RWR-KNN	0.0251	0.0142	0.6005	0.0268
GMNN2CD	0.0444	0.0065	0.1955	0.0118
CD-LNLP	0.0265	0.0078	0.1305	0.0130
KATZHCDA	0.0305	0.0072	0.2665	0.0132
RNMFLP	0.0437	0.0172	0.1409	0.0296
DMFCDA	0.0673	0.0168	0.1792	0.0186
ERFMTDA	**0.1149**	**0.0358**	**0.8596**	**0.0646**

**Note**: AUPR, Pre, Rec, and F1 of different methods in the denovo experiment. The best performance is in bold.

These results demonstrate that ERFMTDA maintains strong predictive power even when encountering completely unseen diseases, highlighting its robustness and generalization capability in de novo prediction scenarios.

### Hyperparameter sensitivity analysis

**Fig 6:**
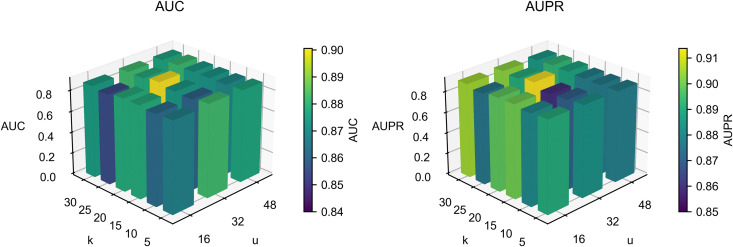
Performance landscapes (AUC and AUPR) under different combinations of *u* and *k.*

To investigate the combined effects of the number of principal components *u* in the association matrix and the top-*k* parameter in the negative sampling module, we conduct a joint hyperparameter sensitivity analysis using AUC and AUPR as evaluation metrics. The results are illustrated in Fig 6.

Overall, the model achieves the best performance when *u* is set to 32 and *k* is set to 20, yielding the highest AUC (0.9006) and AUPR (0.9138). This parameter combination consistently outperforms other settings across both metrics.

When *u* is fixed, the performance with respect to *k* exhibits a clear increasing-then-decreasing trend, particularly when *u* = 32. This behavior indicates that moderate values of *k* allow the negative sampling strategy to effectively avoid false negatives while maintaining sufficient training diversity. In contrast, overly small *k* values result in a weak constraint on negative samples, while excessively large *k* values overly restrict candidate negatives, leading to performance degradation.

When *k* is fixed, a similar pattern is observed across different values of *u*. Compared with smaller (*u* = 16) or larger (*u* = 48) dimensions, *u* = 32 consistently yields superior AUC and AUPR values, suggesting an optimal balance between global structural information preservation and noise suppression.

These results demonstrate that the joint effect of the PCA component number and negative sampling parameters plays a critical role in model performance, and that the proposed parameter settings provide a robust and well-balanced configuration for ERFMTDA.

### Case study

To further demonstrate the practical applicability of ERFMTDA in uncovering biologically meaningful tsRNA–disease associations, we conduct two case studies on representative diseases from different pathological contexts: Diabetic Retinopathy (DR) and Hepatocellular Carcinoma (HCC). DR is a major microvascular complication of diabetes and one of the leading causes of vision impairment worldwide, whereas HCC is one of the most prevalent and lethal malignancies, characterized by complex genetic and epigenetic dysregulation. Investigating tsRNA–disease associations in these two distinct disease settings provides a comprehensive evaluation of the proposed model.

#### Diabetic retinopathy.

We first apply the trained ERFMTDA model to predict candidate tsRNAs associated with Diabetic Retinopathy by ranking all tsRNAs according to their predicted association scores. The top 10 predicted tsRNAs are listed in Table 5, representing the highest-confidence candidates inferred by the model.

**Table 5:** Top 10 predicted tsRNAs that associated with Diabetic Retinopathy.

Rank	tsRNA	Standardized tsRNA ID	Evidence
1	5^′^tiRNA-His-GTG	tDR-1:35-His-GTG-1	PMID: 39513253
2	tiRNA-Val	tDR-1:34-Val-AAC-1-M3	PMID: 35346383
3	5tiRNA-35-PheGAA-8	tDR-1:34-Phe-GAA-8	PMID: 39167377
4	tRF-Glu-CTC	tDR-1:31-Glu-CTC-1-M2	unconfirmed
5	tiRNA-1:33-Gly-GCC-1	tDR-1:33-Gly-GCC-1	unconfirmed
6	tRNA-ValAAC-5	tDR-1:32-Val-AAC-1-M6	unconfirmed
7	tRNA-GlyTCC-5	tDR-1:34-Gly-TCC-2	unconfirmed
8	tiRNA-Val-CAC-001	tDR-1:34-Val-CAC-2	unconfirmed
9	tRF- 1020	tDR-T2:T18-Phe-GAA-1–2	PMID: 36128646
10	tRF-30	tDR-1:30-Gly-GCC-1	PMID: 39001618

Several tsRNAs have previously been reported to play important regulatory roles in the pathogenesis of diabetic retinopathy, providing biological context for our model’s predictions. 5^′^tiRNA-His-GTG has been shown to be markedly upregulated under diabetic stress, where it regulates Müller glial activity, influences endothelial angiogenic responses, and contributes to retinal ganglion cell survival; its inhibition alleviates neurovascular dysfunction by modulating the CYP2E1–19(S)-HETE signaling axis [45]. In addition, tiRNA-Val is significantly increased in DR retinal tissues and in high-glucose–treated endothelial cells, promoting proliferation and inhibiting apoptosis via the Sirt1/Hif-1α pathway; knockdown of tiRNA-Val in vivo improves DR symptoms [46]. Further, tRF-1020 is downregulated in diabetic retinal vessels, where it suppresses endothelial proliferation and tube formation by targeting Wnt signaling; overexpression of tRF-1020 ameliorates vascular leakage and inflammation in vivo [47]. Similarly, tRF-30 is significantly upregulated in both DR models and DR patient vitreous samples, driving pathological neovascularization through the tRF-30/TRIB3/STAT3 signaling pathway [48].

Meanwhile, some predicted tsRNAs (tRF-Glu-CTC, tiRNA-1:33-Gly-GCC-1, tRNA-ValAAC-5, tRNA-GlyTCC-5, tiRNA-Val-CAC-001) have not yet been reported in the context of DR. However, these tsRNAs exhibit potential biological relevance and may serve as novel biomarkers or therapeutic targets, providing promising directions for future experimental validation.

#### Hepatocellular Carcinoma.

To further assess the generalization ability of ERFMTDA across different disease types, we perform an additional case study on Hepatocellular Carcinoma. Using the same trained model, tsRNAs are ranked according to their predicted association scores with HCC, and the top 10 candidates are summarized in Table 6.

**Table 6:** Top 10 predicted tsRNAs that associated with Hepatocellular Carcinoma.

Rank	tsRNA	Standardized tsRNA ID	Evidence
1	tiRNA-Gly-GCC-002	tDR-1:33-Gly-GCC-1	PMID: 39430835
2	CU1276	tDR-55:76-Gly-GCC-2	unconfirmed
3	Gly-tRF	tDR-1:23-Gly-CCC-1-M4	PMID: 34537070
4	tRF-39	tDR-35:73-Asp-GTC-2-M2	PMID: 38455567
5	5^′^-HisGUG	tDR-1:35-His-GTG-1	unconfirmed
6	5^′^-tiRNA-Gln	tDR-1:34-chrM.Gln-TTG	PMID: 36973570
7	tRNA-GluCTC-5	tDR-1:31-Glu-CTC-1-M2	unconfirmed
8	LeuCAG3^′^tsRNA	tDR-55:76-Leu-CAA-1-M5	PMID: 29186115
9	tRF3b-PheGAA-6	tDR-55:76-Phe-GAA-1	unconfirmed
10	tRF3–28-PheGAA-1	tDR-48:75-Phe-GAA-1	unconfirmed

Among the top-ranked tsRNAs predicted for HCC, several have been previously reported to play critical roles in hepatocarcinogenesis. For example, tiRNA-Gly-GCC-002 has been identified as an independent prognostic factor in HCC, with high expression associated with poor patient survival and cell cycle–related functions [49]. Gly-tRF promotes HCC cell migration and malignant progression through the AKT signaling pathway [50]. In addition, tRF-39 is highly expressed in HCC cells, serum, and tumor tissues, where it exerts oncogenic effects through interactions with downstream mRNA targets [51]. Similarly, 5^′^-tiRNA-Gln has been reported to suppress HCC progression by interacting with EIF4A1 and modulating translation through an intramolecular G-quadruplex structure [52].

Meanwhile, several other high-confidence tsRNAs predicted by ERFMTDA, including CU1276, 5^′^-HisGUG, tRNA-GluCTC-5, tRF3b-PheGAA-6, and tRF3–28-PheGAA-1, have not yet been experimentally associated with HCC. These tsRNAs may represent novel regulatory molecules involved in hepatocellular carcinoma and thus constitute promising candidates for future experimental validation.

Overall, the two case studies demonstrate that ERFMTDA can not only successfully recover known disease-associated tsRNAs but also identify novel high-confidence candidates across different disease contexts. These results highlight its potential utility for discovering biologically meaningful tsRNA–disease associations.

## Discussion

In this study, we propose ERFMTDA, a computational framework for predicting tsRNA–disease associations based on Rotative Factorization Machines. Unlike most existing ncRNA–disease association methods that primarily rely on graph topology and predefined similarity measures, ERFMTDA integrates both explicit biological attributes and latent structural information derived from the global association matrix. This design allows the model to better capture the complex and non-linear relationships that underlie tsRNA functions and their involvement in human diseases. A key advantage of ERFMTDA lies in its ability to improve predictive performance in limited data regimes, which is a common challenge in ncRNA–disease association prediction. Due to the relatively recent recognition of tsRNAs as functional regulatory molecules, experimentally validated tsRNA–disease associations remain extremely limited. By integrating global structural features as a complement to explicit biological attributes, ERFMTDA enables the rotative factorization machine to model high-order interactions across heterogeneous feature types, thereby mitigating the adverse effects of data sparsity and enhancing model generalization. In addition, ERFMTDA incorporates a biologically informed negative sampling strategy guided by motif-level sequence similarity. The effectiveness of this strategy can be partially attributed to the sequence-dependent regulatory specificity of tsRNAs, which has been reported in accumulating studies, including miRNA-like seed-based targeting and motif-driven functional divergence [28–31].

Despite its promising performance, this study has several limitations. First, the overall number of experimentally validated tsRNA–disease associations remains small, which inevitably constrains the diversity and coverage of the training data. As more high-quality association data become available, future work will focus on expanding the dataset to further improve model robustness and generalization. Second, although ERFMTDA incorporates multiple sequence-derived features, it does not explicitly model tsRNA secondary structure, which is known to play an important role in RNA stability and molecular interactions [53,54]. Incorporating structural features, such as predicted minimum free energy structures, base-pairing profiles, or secondary structure descriptors, represents a promising direction for future extensions of the framework. These structural representations could be encoded as additional feature embeddings and integrated into the existing feature fusion module alongside biological, semantic, and global structural features, thereby enabling the RFM to capture higher-order interactions between sequence, structure, and disease-related characteristics.

## Conclusion

ERFMTDA provides a robust and flexible computational framework for predicting tsRNA–disease associations by integrating latent structural features, explicit biological attributes, and biologically informed negative sampling within a rotative factorization machine architecture. Extensive evaluations, including cross-validation and de novo testing, demonstrate that ERFMTDA consistently outperforms existing methods. Two case studies on diabetic retinopathy and hepatocellular carcinoma further confirm its ability to identify biologically meaningful tsRNA–disease associations. Overall, this work provides a valuable computational tool for systematically exploring the functional roles of tsRNAs in human diseases and may facilitate future experimental validation and mechanistic investigations.

## Supporting information

S1 Table Performance statistics of ERFMTDA across 10 independent runs under the 5-fold cross-validation setting. (PDF)

S2 Table Performance statistics of ERFMTDA across 10 independent runs under the 10-fold cross-validation setting. (PDF)
