# Supplementary material for: ERFMTDA: Predicting tsRNA–disease associations using an enhanced rotative factorization machine
Source: PLoS Comput Biol. 2026 Aug 18;22(8):e1014594. doi: 10.1371/journal.pcbi.1014594 (PMC13485048; doi:10.1371/journal.pcbi.1014594)
Supplement: Supplementary file 1 [file pcbi.1014594.s001.pdf]

S1 Table. Performance statistics of ERFMTDA across 10 independent runs under the 5-fold cross-validation setting.

**Run seed**	**AUC**	**AUPR**	**Acc**	**Pre**	**Rec**	**F1**
7	0.9122	0.9152	0.8462	0.8258	0.8792	0.8511
42	0.8845	0.9023	0.8379	0.8118	0.8823	0.8453
436	0.8759	0.8915	0.8183	0.8096	0.8368	0.8218
194	0.8846	0.8939	0.8150	0.7917	0.8562	0.8218
543	0.8802	0.8914	0.8200	0.7970	0.8596	0.8269
2083	0.8847	0.8850	0.8167	0.7866	0.8727	0.8267
3001	0.8839	0.8917	0.8413	0.8205	0.8758	0.8470
2048	0.8839	0.8962	0.8134	0.7990	0.8399	0.8186
130	0.8755	0.8914	0.8150	0.7972	0.8464	0.8209
1983	0.9006	0.9138	0.8348	0.8205	0.8628	0.8398
Average	0.8869±0.0107	0.8972±0.0096	0.8259±0.0120	0.8060±0.0128	0.8612±0.0155	0.8320±0.0118

Note: AUC, AUPR, Acc, Pre, Rec, and F1 of different runs in the 5-fold cross validation experiment.
