# Supplementary material for: ERFMTDA: Predicting tsRNA–disease associations using an enhanced rotative factorization machine
Source: PLoS Comput Biol. 2026 Aug 18;22(8):e1014594. doi: 10.1371/journal.pcbi.1014594 (PMC13485048; doi:10.1371/journal.pcbi.1014594)
Supplement: Supplementary file 2 [file pcbi.1014594.s002.pdf]

S2 Table. Performance statistics of ERFMTDA across 10 independent runs under the 10-fold cross-validation setting.

Run seed	AUC	AUPR	Acc	Pre	Rec	F1
7	0.9049	0.9183	0.8379	0.8188	0.8726	0.8435
42	0.8935	0.8913	0.8380	0.8143	0.8824	0.8458
436	0.8960	0.9121	0.8281	0.8137	0.8627	0.8350
194	0.9178	0.9249	0.8412	0.8068	0.8983	0.8492
543	0.8925	0.9018	0.8444	0.8373	0.8630	0.8483
2083	0.8958	0.8923	0.8265	0.7995	0.8787	0.8357
3001	0.8844	0.9014	0.8102	0.7913	0.8471	0.8143
2048	0.8884	0.8995	0.8135	0.7905	0.8562	0.8212
130	0.8847	0.9031	0.8086	0.7913	0.8428	0.8150
1983	0.9011	0.9163	0.8495	0.8441	0.8691	0.8545
Average	0.8959±0.0096	0.9061±0.0107	0.8298±0.0141	0.8108±0.0179	0.8673±0.0159	0.8363±0.0140

Note: AUC, AUPR, Acc, Pre, Rec, and F1 of different runs in the 10-fold cross validation experiment.
